# Self‐Report versus Clinician Examination in Early Parkinson's Disease

**DOI:** 10.1002/mds.28884

**Published:** 2021-12-13

**Authors:** Sheida Zolfaghari, Alessandra E. Thomann, Natalia Lewandowski, Dylan Trundell, Florian Lipsmeier, Gennaro Pagano, Kirsten I. Taylor, Ronald B. Postuma

**Affiliations:** ^1^ Integrated Program in Neuroscience McGill University Montreal Quebec Canada; ^2^ Research Institute of the McGill University Health Centre Montreal Quebec Canada; ^3^ Roche Pharma Research and Early Development, Neuroscience and Rare Diseases Discovery and Translational Area Basel Switzerland; ^4^ Faculty of Science McGill University Montreal Quebec Canada; ^5^ Roche Products Limited Welwyn Garden City United Kingdom; ^6^ Roche Pharma Research and Early Development, pRED Informatics, Roche Innovation Center Basel Switzerland; ^7^ King's College London London United Kingdom; ^8^ Department of Neurology and Neurosurgery McGill University Montreal Quebec Canada; ^9^ Centre for Advanced Research in Sleep Medicine Hôpital du Sacré‐Coeur de Montréal Montreal Quebec Canada

**Keywords:** Movement Disorder Society Unified Parkinson's Disease Rating Scale, motor symptoms, motor signs, Parkinson's disease

## Abstract

**Background:**

Evaluating the discrepancies between patient‐reported measures and clinician examination has implications for formulating individual treatment regimens.

**Objective:**

This study investigated the association between health outcomes and level of self‐reported motor‐related function impairment relative to clinician‐examined motor signs.

**Methods:**

Recently diagnosed PD patients were evaluated using the Parkinson's Progression Marker Initiative (PPMI, N = 420) and the PASADENA phase II clinical trial (N = 316). We calculated the average normalized difference between each participant's part II and III MDS‐UPDRS (Movement Disorder Society Unified Parkinson's Disease Rating Scale) scores. Individuals with score differences <25th or >75th percentiles were labeled as low‐ and high‐self‐reporters, respectively (those between ranges were labeled intermediate‐self‐reporters). We compared a wide range of clinical/biomarker readouts among these three groups, using Kruskal–Wallis nonparametric and Pearson's χ^2^ tests. Spearman's correlations were tested for associations between MDS‐UPDRS subscales.

**Results:**

In both cohorts, high‐self‐reporters reported the largest impairment/symptom experience for most motor and nonmotor patient‐reported variables. By contrast, these high‐self‐reporters were similar to or less impaired on clinician‐examined and biomarker measures. Patient‐reported nonmotor symptoms on MDS‐UPDRS part IB showed the strongest positive correlation with self‐reported motor‐related impairment (PPMI r_s_ = 0.54, PASADENA r_s_ = 0.52). This correlation was numerically stronger than the part II and clinician‐examined MDS‐UPDRS part III correlation (PPMI r_s_ = 0.38, PASADENA r_s_ = 0.28).

**Conclusion:**

Self‐reported motor‐related impairments reflect not only motor signs/symptoms but also other self‐reported nonmotor measures. This may indicate (1) a direct impact of nonmotor symptoms on motor‐related functioning and/or (2) the existence of general response tendencies in how patients self‐rate symptoms. Our findings suggest further investigation into the suitability of MDS‐UPDRS II to assess motor‐related impairments. © 2021 The Authors. *Movement Disorders* published by Wiley Periodicals LLC on behalf of International Parkinson and Movement Disorder Society

When treating Parkinson's disease (PD), the decisions about when to prescribe symptomatic treatments and what agents to use are unique to each person.^1^ To formulate the proper treatment regimen, physicians rely primarily on patient‐reported symptoms supplemented by expert examination. Moreover, when new drugs are tested, the Food and Drug Administration (FDA) highlights the primacy of patient‐reported scales complemented by clinician examinations to achieve the most meaningful outcomes for patients.

In PD, one of the most commonly used scales to assess motor impairment is the Movement Disorder Society Unified Parkinson's Disease Rating Scale (MDS‐UPDRS), which includes a patient‐reported questionnaire to assess motor aspects of experiences of daily living (ie, part II) and a clinician‐examined motor examination (ie, part III). Because both parts are intended to assess aspects of motor impairment in PD, one would expect a close relationship between these subscales. However, it has previously been shown that self‐reported measures may be associated with comorbid conditions such as anxiety, depression,^2^, ^3^, ^4^, ^5^ or cognitive deficits.^6^, ^7^ In particular, Weintraub and colleagues^8^ suggested that nonmotor symptoms of PD may impact patients' endorsement of motor problems in daily life, supported by other studies demonstrating that anxiety and depression are strongly related to self‐reported quality of life and motor severity.^9^ Furthermore, unawareness or underrecognition of certain symptoms has been well documented in PD.^10^, ^11^, ^12^, ^13^, ^14^ Other differences between patient‐reported and clinician‐examined measures may result from differences in the scale design itself. For instance, whereas MDS‐UPDRS III is focused on motor examination only, MDS‐UPDRS II was developed to assess the impact of motor symptoms (eg, tremor) on daily activities (eg, eating tasks).^15^ Although MDS‐UPDRS III cannot be considered objective, the standardized training for administration and well‐defined and differentiated response options help reduce the potential for subjective differences between or within raters. In comparison, MDS‐UPDRS II provides a more subjective interpretation of the experienced impact of motor impairment that depends on each individual patient's reference system.

Given the extensive use of MDS‐UPDRS in clinical practice and clinical trials, we aimed to investigate the relationship between MDS‐UPDRS parts II and III specifically to identify (1) whether and to what extent discrepancies between self‐assessed and clinician‐examined measures exist and (2) how these differences are associated with demographic and clinical characteristics. This study analyzed data from individuals with early PD participating in two large studies: the Parkinson's Progression Marker Initiative (PPMI) observational clinical study and the phase II clinical trial PASADENA. Gaining a better understanding of potential differences between patients' self‐reports and clinical motor examinations has implications for formulating individual treatment regimens and evaluating treatment success in clinical drug development. The findings of this study thereby may help to ensure that individuals with PD receive proper treatment for their needs.

## Patients and Methods

### Study Population

**FIG. 1:**
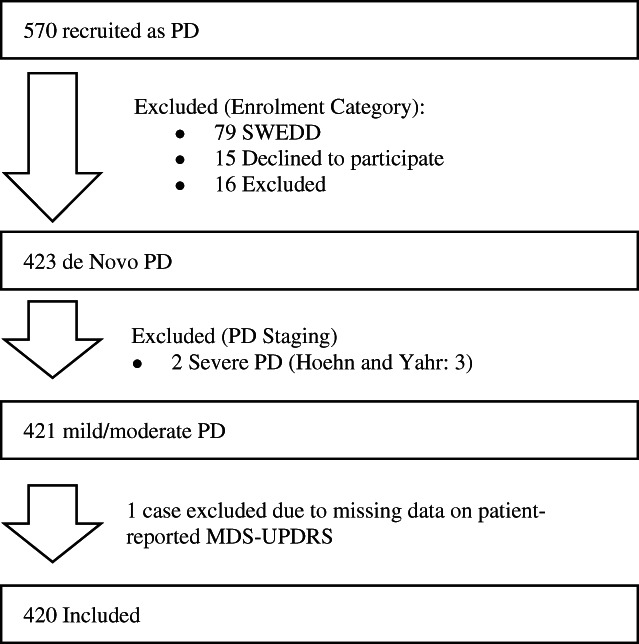
Inclusion chart for PPMI: among the de novo PD patients of PPMI, we selected those with Hoehn and Yahr stages 1 and 2 and those with available data on patient‐reported and clinician‐reported MDS‐UPDRS. PPMI, Parkinson's Progression Markers Initiative; PD, Parkinson's disease; SWEDD, Scans Without Evidence of Dopaminergic Deficit; MDS‐UPDRS, Movement Disorder Society Unified Parkinson Disease Rating Scale.

PPMI is an international multicentric cohort study to explore the cause and natural history of PD through longitudinal investigation of the progression of PD biomarkers.^16^ The PPMI protocol and eligibility criteria are available elsewhere.^16^ The PPMI protocol was reviewed and approved by the Institutional Review Board and the Independent Ethics Committee (IRB/IEC) at each center. Written informed consent was obtained from all participants. For the present analyses and among the 423 individuals with de novo diagnosed PD in the PPMI study, we selected 420 with baseline Hoehn and Yahr (HY) stages 1 and 2, who had no missing baseline data on MDS‐UPDRS scale (Fig. 1). We used the baseline data (downloaded in June 2019) at which time participants were not taking any symptomatic PD medications.

PASADENA is a phase II clinical trial (NCT03100149) sponsored by F. Hoffmann‐La Roche Ltd. The study was approved by the IRB or IEC. Further information on the protocol and eligibility criteria can be found at https://clinicaltrials.gov/ct2/show/NCT03100149 and in Pagano et al.^17^ At baseline, data from 316 individuals with recently diagnosed PD (ie, disease duration <2 years) were available. All patients were either treatment naïve or treated with a stable dose of monoamine oxidase‐B inhibitors (36.4%) and had HY stages 1 and 2, with a diagnosis of PD confirmed by the dopamine transporter single‐photon emission computed tomography (DaT‐SPECT). Only baseline data were considered for the present analysis; therefore, no drug effects were investigated.

### Measurements

#### Self‐Reported Motor‐Related Impact and Clinician‐Examined Motor Signs

We investigated MDS‐UPDRS II as the self‐reported measurement of motor‐related impact experienced by individuals with PD in their daily lives and MDS‐UPDRS III as the clinician‐examined measurement of motor signs.

Thirteen items in MDS‐UPDRS II and 33 items in MDS‐UPDRS III are each ranked on a five‐point Likert scale (0 = normal, 1 = slight, 2 = mild, 3 = moderate, and 4 = severe). The MDS‐UPDRS II score ranges from 0 to 52, and MDS‐UPDRS III score ranges from 0 to 132. To facilitate comparisons of relative scores on parts II and III, each score was normalized by the number of possible items on each scale (ie, part II score/13 items and part III score/33 items). Thus, the normalized score represented the average severity rating (0–4 points) per item. The difference in normalized scores was then generated per participant (ie, *individual normalized MDS‐UPDRS II score* – *individual normalized MDS‐UPDRS III score*). The resulting difference score represented the average severity of part II relative to part III items, whereby negative values indicated higher average severity scores on part III than part II and vice versa.

The categorization in groups of high‐, intermediate‐, and low‐self‐reporters was done separately for each cohort (ie, PPMI vs. PASADENA). Each participant was categorized by their difference score according to their position in the respective frequency distribution. Individuals with a difference score below the 25th percentile were labeled as low‐self‐reporters (ie, self‐report ratings relatively lower than clinician ratings) and those above the 75th percentile as high‐self‐reporters (ie, self‐report ratings relatively higher than clinician ratings). Individuals with score differences between the 25th and 75th percentiles were labeled as intermediate‐self‐reporters. This approach does not decide the “true” severity of motor symptoms; neither clinician‐examined nor self‐report markers were considered the gold standard. Rather, participants were compared with each other in their overall relative self‐ versus examiner ratings.

#### Sociodemographic and Disease Characteristics, Other Patient‐Reported and Clinician‐Examined Measures, Cognitive Outcomes, and Biomarkers

We compared low‐ versus intermediate‐ versus high‐self‐reporters for (1) sociodemographic characteristics, (2) disease characteristics, (3) patient‐reported measures, (4) clinician‐reported measures based on patient‐reported information, (5) other clinician‐examined and objective measures, (6) cognitive test results (PPMI only), (7) DaT‐SPECT (PPMI only), and (8) cerebrospinal fluid (CSF) biomarkers (PPMI only).

The variables in each category and their classification and scoring are presented in detail in Appendix S1.

### Statistical Analyses

Descriptive results comprised means and standard deviations for continuous variables and frequencies and percentages for categorical variables. Kruskal–Wallis nonparametric tests compared low‐, intermediate‐, and high‐self‐reporter scores on continuous variables. Categorical variables were analyzed using Pearson's χ^2^ test. Because the groups were assigned according to individual place in the frequency distribution in the respective population and the borders between intermediate‐ and low‐/high‐self‐reporters were somewhat arbitrary, the primary focus of this study was to compare the extreme ends of the distribution. Therefore, Cohen's d was used to quantify the effect sizes of differences between high‐ and low‐self‐reporters for continuous variables and Cramer's V for categorical variables.

A significance level of α = 0.05 was used for all statistical tests, not corrected for multiple comparisons.

### Secondary Analysis

To obtain further insights into the potential intercorrelation between the patient‐reported and clinician‐examined scales, Spearman's correlations tested for associations between MDS‐UPDRS parts II and III and for their respective associations with parts IA and IB. Because parts II and III are both intended to assess motor‐related impairment, we expected a high correlation between these two scores. Moreover, to identify whether and which factors are associated with self‐reported and clinician‐examined ratings, we used multivariable linear regression models to predict MDS‐UPDRS parts II and III, respectively, with parts IA, IB, and II or III, age, and sex as predictor variables. Variables were scaled before being entered into the regression model.

## Results

### Parkinson's Progression Marker Initiative

The 420 individuals with PD from the PPMI cohort had a mean baseline age of 61.6 ± 9.7 years, and 65.7% were men. One hundred and three participants were categorized as low‐self‐reporters, 214 as intermediate‐self‐reporters, and 103 as high‐self‐reporters. There were no differences in age, sex, and other sociodemographic variables between the three groups (all *P* > 0.05; Table 1). Age of onset, duration of PD, the most affected side at the onset of disease, and common genetic variants (β‐glucocerebrosidase and leucine‐rich repeat kinase 2) were similar between these groups (all *P* > 0.05; Table 1).

**TABLE 1:** Comparison of sociodemographic characteristics among low‐self‐reporters, intermediate‐self‐reporters, and high‐self‐reporters based on motor aspects of Parkinson's disease

			PPMI N = 420					PASADENA N = 316				
			Low‐self‐reporterN = 103	Intermediate‐self‐reporter N = 214	High‐self‐reporter N = 103	*P*‐value	Cohen's d effect size between low‐ and high‐self‐reporters	Low‐self‐reporter N = 80	Intermediate‐self‐reporter N = 157	High‐self‐reporter N = 79	*P*‐value	Cohen's d effect size between low‐ and high‐self‐reporters
Sociodemographic characteristics	Male sex—% (n)		66.0 (68)	66.4 (142)	64.1 (66)	0.9	0.20^*^	68.8 (55)	65.0 (102)	70.9 (56)	0.6	0.05^*^
	Age (y)		62.6 ± 8.4	61.4 ± 9.9	61.2 ± 10.5	0.6	0.15	60.2 ± 8.8	59.6 ± 8.7	59.6 ± 10.3	0.9	0.06
	White race—% (n)		96.1 (99)	89.7 (192)	94.2 (97)	0.5	0.05^*^	88.8 (71)	82.8 (130)	78.5 (62)	0.2	0.1^*^
	Education (y)		15.8 ± 3.2	15.5 ± 2.9	15.4 ± 3.0	0.6	0.13	16.5 ± 4.0	16.5 ± 3.3	15.9 ± 4.8	0.14	0.14
	BMI (kg/m^2^)		27.9 ± 5.0	26.8 ± 4.5	27.1 ± 4.5	0.1	0.17	25.4 ± 3.2	25.5 ± 3.6	25.2 ± 3.3	1	0.06
	Age of onset (y)		60.2 ± 9.0	59.6 ± 10.0	59.0 ± 10.6	0.8	0.12	59.2 ± 8.8	58.8 ± 8.7	58.8 ± 10.3	0.9	0.04
	Duration of disease (mo)		6.4 ± 5.7	6.7 ± 6.9	7.0 ± 6.7	0.8	0.09	11.4 ± 7.5	9.6 ± 5.9	9.8 ± 6.5	0.3	0.23
	Right‐handed—% (n)		90.3 (93)	88.3 (189)	87.4 (90)	1	0.05^*^	93.8 (75)	86.6 (136)	93.7 (74)	0.1	0.12^*^
	Right side most affected at onset—% (n)		52.4 (54)	54.2 (116)	62.1 (64)	0.4	0.11^*^	48.8 (39)	57.3 (90)	58.2 (46)	0.4	0.08^*^
	First‐degree family with PD—% (n)		15.5 (16)	10.7 (23)	14.7 (15)	0.5	0.08^*^	–	–	–	–	–
	Common genetic variants—% (n)											
		GBA	9.7 (10)	10.7 (23)	7.8 (8)	0.7	0.03^*^	–	–	–	–	–
		LRRK2	30.1 (31)	26.2 (56)	28.2 (29)	0.8	0.02^*^	–	–	–	–	–
	Prior MAO‐B inhibitor therapy—% (n)		–	–	–	**–**	–	32.5 (26)	35.0 (55)	43.0 (34)	0.3	0.08^*^

^*^ For categorical variables, Cramer V, instead of Cohen's d, was calculated to estimate the effect size.

Abbreviations: PPMI, Parkinson's Progression Markers Initiative; BMI, body mass index; PD, Parkinson's disease; GBA, β‐glucocerebrosidase; LRRK2, leucine‐rich repeat kinase 2; MAO‐B, monoamine oxidase‐B.

High‐, intermediate‐, and low‐self‐reporters differed on almost all patient‐reported scales, except for the Questionnaire for Impulsive‐Compulsive Disorders in Parkinson's Disease (QUIP; *P* = 0.1).

On MDS‐UPDRS part I, high‐self‐reporters reported more nonmotor symptoms and associated difficulties in daily life compared to low‐ and intermediate‐self‐reporters (4.4 ± 2.8 vs. 5.1 ± 3.8 vs. 7.7 ± 4.8, in low‐, intermediate‐, and high‐self‐reporters, respectively, *P* < 0.001). The same pattern remained when subdivided into MDS‐UPDRS IA and IB (part IA: 1.0 ± 1.1 vs. 1.1 ± 1.4 vs. 1.7 ± 2.1, in low‐, intermediate‐, and high‐self‐reporters, respectively, *P* = 0.035; part IB: 3.4 ± 2.4 vs. 4.0 ± 3.0 vs. 6.0 ± 3.6, in low‐, intermediate‐, and high‐self‐reporters, respectively, *P* < 0.001). On MDS‐UPDRS part I item level, most individual questions were rated higher among high‐self‐reporters than among the other two groups (Table 2).

**TABLE 2:** Comparison of patient‐reported measures, clinician‐based exams, DaT‐SPECT scan, and CSF biomarkers among low‐self‐reporters, intermediate‐self‐reporters, and high‐self‐reporters based on motor aspects of Parkinson's disease

			PPMI N = 420					PASADENA N = 316				
			Low‐self‐reporter N = 103	Intermediate‐self‐reporter N = 214	High‐self‐reporter N = 103	*P*‐value	Cohen's d effect size between low‐ and high‐self‐reporters	Low‐self‐reporter N = 80	Intermediate‐self‐reporter N = 157	High‐self‐reporter N = 79	*P*‐value	Cohen's d effect size between low‐ and high‐self‐reporters
Patient‐reported measures	**MDS‐UPDRS part IB (score)**		3.4 ± 2.4	4.0 ± 3.0	6.0 ± 3.6	**<0.001** ^b^ ^,^ ^c^	0.86	2.1 ± 1.8	3.3 ± 2.4	5.2 ± 3.4	**<0.001** ^a^ ^,^ ^b^ ^,^ ^c^	1.14
		Sleep problems (night)	0.8 ± 0.9	0.8 ± 1.0	1.2 ± 1.2	**0.020** ^b^ ^,^ ^c^	0.34	0.5 ± 0.7	0.8 ± 0.9	1.3 ± 1.0	**<0.0001** ^a^ ^,^ ^b^ ^,^ ^c^	0.93
		Daytime sleepiness	0.6 ± 0.8	0.6 ± 0.8	0.9 ± 0.8	**0.001** ^b^ ^,^ ^c^	0.39	0.4 ± 0.6	0.5 ± 0.7	0.8 ± 0.8	**0.0004** ^a^ ^,^ ^b^ ^,^ ^c^	0.57
		Pain and other sensations	0.5 ± 0.6	0.6 ± 0.8	1.1 ± 1.0	**<0.001** ^b^ ^,^ ^c^	0.69	0.3 ± 0.5	0.5 ± 0.6	0.8 ± 0.6	**0.0001** ^a^ ^,^ ^b^ ^,^ ^c^	0.91
		Urinary problems	0.5 ± 0.7	0.7 ± 0.9	0.7 ± 0.8	**0.007** ^a^ ^,^ ^b^	0.37	0.4 ± 0.6	0.4 ± 0.7	0.8 ± 0.9	**0.001** ^b^ ^,^ ^c^	0.52
		Constipation problems	0.3 ± 0.6	0.4 ± 0.7	0.6 ± 0.8	0.1	0.32	0.1 ± 0.3	0.4 ± 0.7	0.5 ± 0.7	**0.001** ^a^ ^,^ ^b^	0.74
		Light‐headedness on standing	0.2 ± 0.5	0.3 ± 0.5	0.4 ± 0.7	**0.011** ^b^ ^,^ ^c^	0.35	0.1 ± 0.3	0.2 ± 0.4	0.3 ± 0.5	**0.01** ^b^ ^,^ ^c^	0.49
		Fatigue	0.4 ± 0.6	0.5 ± 0.7	1.1 ± 1.0	**<0.001** ^b^ ^,^ ^c^	0.86	0.3 ± 0.5	0.5 ± 0.7	0.7 ± 0.8	**0.0007** ^b^ ^,^ ^c^	0.60
	**MDS‐UPDRS part II (score)**		3.5 ± 2.5	4.9 ± 3.1	10.4 ± 4.1	**<0.001** ^a^ ^,^ ^b^ ^,^ ^c^	2.07	2.9 ± 2.3	4.4 ± 2.9	9.7 ± 4.1	**<0.001** ^a^ ^,^ ^b^ ^,^ ^c^	2.05
	STAI/HADS‐A (score)		64.4 ± 18.8	63.9 ± 17.4	69.6 ± 19.3	**0.022** ^b^ ^,^ ^c^	0.27	4.2 ± 2.7	4.7 ± 3.0	5.5 ± 3.6	**0.03** ^b^ ^,^ ^c^	0.41
	GDS/ HADS‐D (score)		2.1 ± 2.4	2.2 ± 2.3	3.0 ± 2.7	**0.005** ^b^ ^,^ ^c^	0.35	3.3 ± 3.1	3.7 ± 2.9	5.7 ± 3.7	**<0.001** ^a^ ^,^ ^b^ ^,^ ^c^	0.70
		Depressed mood (GDS ≥ 5/HADS‐D ≥ 11)—% (n)	11.7 (12)	13.1 (28)	18.4 (19)	0.3	0.10*	0.04 (6)	0.03 (10)	0.12 (23)	**0.01**	0.18
	Epworth sleepiness scale (score)		5.2 ± 3.0	5.6 ± 3.5	6.9 ± 3.7	**0.001** ^b^ ^,^ ^c^	0.52	–	–	–	–	–
	QUIP (score)		0.2 ± 0.5	0.3 ± 0.6	0.4 ± 0.8	0.1	0.28	–	–	–	–	–
	RBDSQ (score)		3.2 ± 2.2	4.1 ± 2.7	5.1 ± 2.9	**<0.001** ^a^ ^,^ ^b^ ^,^ ^c^	0.71	3.1 ± 2.7	3.3 ± 2.5	4.2 ± 2.9	**0.005** ^b^ ^,^ ^c^	0.39
	SCOPA‐AUT (score)		7.2 ± 4.3	9.3 ± 6.3	12.3 ± 6.6	**<0.001** ^a^ ^,^ ^b^ ^,^ ^c^	0.90	6.4 ± 4.3	7.6 ± 5.1	10.5 ± 7.2	**0.0005** ^b^ ^,^ ^c^	0.69
		Gastrointestinal dysfunction (score)	1.7 ± 1.7	1.9 ± 1.9	3.2 ± 2.4	**<0.001** ^b^ ^,^ ^c^	0.76	–	–	–	–	–
		Urinary dysfunction (score)	3.5 ± 2.4	4.3 ± 3.1	4.8 ± 3.2	**0.004** ^a^ ^,^ ^b^	0.47	–	–	–	–	–
		Cardiovascular dysfunction (score)	0.3 ± 0.6	0.4 ± 0.7	0.7 ± 0.9	**0.001** ^b^ ^,^ ^c^	0.50	–	–	–	–	–
		Thermoregulatory dysfunction (score)	0.8 ± 1.1	1.1 ± 1.3	1.7 ± 1.7	**<0.001** ^b^ ^,^ ^c^	0.61	–	–	–	–	–
		Pupillomotor dysfunction (score)	0.3 ± 0.6	0.4 ± 0.6	0.6 ± 0.7	**0.001** ^b^ ^,^ ^c^	0.46	–	–	–	–	–
		Sexual dysfunction (score)	0.7 ± 1.2	1.2 ± 1.6	1.3 ± 1.6	**0.020** ^a^ ^,^ ^b^	0.40	–	–	–	–	–
	PDQ‐39 Index Score		–	–	–	–	–	7.3 ± 5.1	9.2 ± 6.9	13.5 ± 7.2	**<0.0001** ^a^ ^,^ ^b^ ^,^ ^c^	0.99
		ADL	–	–	–	–	–	6.4 ± 8.5	8.3 ± 10.8	15.3 ± 11.6	**<0.0001** ^b^ ^,^ ^c^	0.88
		Bodily discomfort	–	–	–	–	–	13.6 ± 14.5	17.9 ± 17.9	22.3 ± 17.3	**0.0068** ^b^ ^,^ ^c^	0.55
		Cognition	–	–	–	–	–	6.1 ± 10.5	8.9 ± 10.7	13.1 ± 13.8	**0.0013** ^a^ ^,^ ^b^ ^,^ ^c^	0.57
		Communication	–	–	–	–	–	2.6 ± 5.9	5.7 ± 9.8	12.5 ± 14.1	**<0.0001** ^a^ ^,^ ^b^ ^,^ ^c^	0.92
		Emotional well‐being	–	–	–	–	–	11.0 ± 9.9	13.1 ± 12.7	18.5 ± 13.7	**0.0026** ^b^ ^,^ ^c^	0.63
		Mobility	–	–	–	–	–	2.5 ± 3.9	4.1 ± 6.8	8.8 ± 11.6	**0.0001** ^a^ ^,^ ^b^ ^,^ ^c^	0.73
		Social support	–	–	–	–	–	1.7 ± 4.9	2.5 ± 6.2	2.2 ± 5.3	0.56	0.1
		Stigma	–	–	–	–	–	14.4 ± 15.6	13.2 ± 14.7	15.1 ± 17.5	0.87	0.04
Examination Measures	Hoehn and Yahr stage		1.8 ± 0.4	1.5 ± 0.5	1.5 ± 0.5	**<0.001** ^a^ ^,^ ^b^	0.58	2.0 ± 0.2	1.7 ± 0.5	1.6 ± 0.5	**0.0005**	1.05
	**MDS‐UPDRS part III (score)**		28.1 ± 8.0	18.6 ± 8.0	18.4 ± 7.4	**<0.001** ^a^ ^,^ ^b^	1.26	29.1 ± 6.1	19.4 ± 8.0	17.8 ± 8.9	**<0.001** ^a^ ^,^ ^b^	1.48
		Tremor score	5.8 ± 3.5	4.1 ± 2.9	3.5 ± 2.9	**<0.001** ^a^ ^,^ ^b^	0.73	–	–	–	–	–
		Rigidity score	5.2 ± 2.8	3.4 ± 2.5	3.2 ± 2.4	**<0.001** ^a^ ^,^ ^b^	0.78	–	–	–	–	–
		Bradykinesia score	13.4 ± 5.3	8.3 ± 5.0	8.5 ± 4.4	**<0.001** ^a^ ^,^ ^b^	1.01	–	–	–	–	–
Clinician report based on patient‐reported information	**MDS‐UPDRS part I (score)**		4.4 ± 2.8	5.1 ± 3.8	7.7 ± 4.8	**<0.001** ^b^ ^,^ ^c^	0.84	2.8 ± 2.2	4.3 ± 3.3	7.1 ± 4.8	**<0.001** ^a^ ^,^ ^b^ ^,^ ^c^	1.15
	**MDS‐UPDRS part IA (score)**		1.0 ± 1.1	1.1 ± 1.4	1.7 ± 2.1	**0.035** ^b^ ^,^ ^c^	0.41	0.7 ± 1.0	1.0 ± 1.3	1.9 ± 2.3	**<0.001** ^a^ ^,^ ^b^ ^,^ ^c^	0.68
		Cognitive impairment	0.2 ± 0.5	0.3 ± 0.5	0.4 ± 0.6	0.3	0.25	0.1 ± 0.3	0.2 ± 0.5	0.4 ± 0.7	**0.0005** ^a^ ^,^ ^b^ ^,^ ^c^	0.56
		Hallucinations and psychosis	0.0 ± 0.2	0.0 ± 0.2	0.0 ± 0.1	0.3	0.23	0.0 ± 0.0	0.0 ± 0.1	0.0 ± 0.2	0.4	0.00
		Depressed mood	0.2 ± 0.4	0.3 ± 0.5	0.4 ± 0.7	0.1	0.36	0.1 ± 0.3	0.1 ± 0.4	0.4 ± 0.7	**0.0001** ^b^ ^,^ ^c^	0.56
		Anxious mood	0.4 ± 0.5	0.4 ± 0.6	0.5 ± 0.7	0.5	0.17	0.4 ± 0.6	0.5 ± 0.7	0.7 ± 0.7	0.7^b^	0.46
		Apathy	0.1 ± 0.4	0.1 ± 0.4	0.4 ± 0.7	**<0.001** ^b^ ^,^ ^c^	0.44	0.1 ± 0.3	0.1 ± 0.4	0.4 ± 0.7	**0.0005** ^b^ ^,^ ^c^	0.56
		Features of DDS	0.0 ± 0.2	0.0 ± 0.1	0.0 ± 0.2	0.3	0.10	0.0 ± 0.0	0.0 ± 0.0	0.1 ± 0.3	**0.01** ^b^ ^,^ ^c^	0.47
	Modified Schwab and England ADL (score)		92.8 ± 5.5	94.2 ± 5.6	91.4 ± 6.5	**<0.001** ^a^ ^,^ ^c^	0.24	92.8 ± 5.9	93.7 ± 6.2	90.0 ± 5.8	**<0.001** ^b^ ^,^ ^c^	0.48
Objective measures	UPSIT (score)		21.8 ± 7.7	21.7 ± 7.9	24.1 ± 9.1	**0.044** ^b^ ^,^ ^c^	0.28	–	–	–	–	–
		Normosmia—% (n)	6.8 (7)	6.4 (13)	17.5 (18)	**0.018** ^c^	0.16*	–	–	–	–	–
		Hyposmia—(n)	57.3 (59)	58.4 (125)	49.5 (51)			–	–	–	–	–
		Anosmia—(n)	35.9 (37)	35.5 (76)	33.0 (34)			–	–	–	–	–
	Drop in systolic blood pressure (mmHg)		5.4 ± 12.4	4.8 ± 13.2	3.7 ± 12.0	0.6	0.14	–	–	–	–	–
	Cognitive tests											
		MoCA (score)	26.7 ± 2.4	27.4 ± 2.2	27.1 ± 2.4	0.1	0.15	–	–	–	–	–
		BJLOT (score)	11.8 ± 2.9	12.2 ± 3.0	12.0 ± 2.8	0.4	0.07	–	–	–	–	–
		HVLT‐R: total recall (T‐score)	44.2 ± 11.0	46.3 ± 10.6	45.6 ± 10.9	0.2	0.13	–	–	–	–	–
		HVLT‐R: delayed recall (T‐score)	43.6 ± 11.7	44.8 ± 11.1	45.9 ± 10.0	0.4	0.21	–	–	–	–	–
		HVLT‐R: retention (T‐score)	46.3 ± 11.9	46.8 ± 11.7	48.6 ± 10.5	0.6	0.20	–	–	–	–	–
		HVLT‐R: recognition discrimination index (T‐score)	43.4 ± 11.0	45.5 ± 11.0	45.2 ± 11.7	0.2	0.16	–	–	–	–	–
		Letter number sequencing (score)	11.3 ± 2.7	11.5 ± 2.6	11.6 ± 2.8	0.7	0.09	–	–	–	–	–
		Semantic fluency test (score)	47.7 ± 10.5	49.0 ± 11.7	48.7 ± 12.6	0.9	0.09	–	–	–	–	–
		Symbol digit modality test (T‐score)	45.0 ± 10.5	45.3 ± 9.0	44.4 ± 8.3	0.7	0.06	–	–	–	–	–
Brain imaging	DaT‐SPECT SBR							–	–	–	–	–
		Lowest caudate	1.9 ± 0.5	1.8 ± 0.5	1.8 ± 0.6	0.2	0.06	–	–	–	–	–
		Lowest putamen	0.7 ± 0.2	0.7 ± 0.2	0.7 ± 0.3	0.7	0.09	–	–	–	–	–
		Lowest striatum	2.5 ± 0.7	2.5 ± 0.7	2.5 ± 0.9	0.2	0.01	–	–	–	–	–
CSF biomarkers	Amyloid β 1–42 (pg/mL)		889.4 ± 369.2	926.0 ± 429.0	896.8 ± 416.9	0.7	0.02	–	–	–	–	–
	α‐Synuclein (pg/mL)		1500.6 ± 558.9	1531.1 ± 724.2	1458.6 ± 650.7	0.4	0.07	–	–	–	–	–
	Total‐tau (t‐tau) (pg/mL)		164.9 ± 53.3	170.8 ± 57.4	171.1 ± 59.9	0.7	0.11	–	–	–	–	–
	Phosphorylated‐tau (p‐tau) (pg/mL)		14.5 ± 4.9	14.9 ± 5.2	15.2 ± 5.7	0.8	0.13	–	–	–	–	–

Numbers in bold are *P*‐values <0.05. Results were not corrected for multiple comparisons.

^*^ For this cell, due to its categorical nature, Cramer V is calculated to measure the effect size, instead of Cohen's d.

^a^ Statistically significant difference between low‐ and intermediate‐self‐reporters.

^b^ Statistically significant difference between low‐ and high‐self‐reporters.

^c^ Statistically significant difference between intermediate‐ and high‐self‐reporters.

Abbreviations: DaT‐SPECT, dopamine transporter single‐photon emission computerized tomography; CSF, cerebrospinal fluid; PPMI, Parkinson's Progression Markers Initiative; MDS‐UPDRS, Movement Disorder Society‐Sponsored Revision of the Unified Parkinson's Disease Rating Scale; STAI, State–Trait Anxiety Inventory; HADS‐A, Hospital Anxiety and Depression Scale‐Anxiety section; GDS, Geriatric Depression Scale; HADS‐D, Hospital Anxiety and Depression Scale‐Depression section; PD, Parkinson's Disease; QUIP, Questionnaire for Impulsive‐Compulsive Disorders in Parkinson's Disease; RBDSQ, REM Sleep Behavior Disorder Screening Questionnaire; SCOPA‐AUT, Scales for Outcomes in Parkinson's Disease‐Autonomic Questionnaire; PDQ‐39, Parkinson's Disease Questionnaire‐39; ADL, activities of daily living; DDS, Dopamine Dysregulation Syndrome; UPSIT, University of Pennsylvania Smell Identification Test; MoCA, Montreal Cognitive Assessment; BJLOT, Benton Judgment of Line Orientation Test; HVLT‐R, Hopkins Verbal Learning Test‐Revised; SBR, Specific Binding Ratio.

Patient‐reported measures such as anxiety, depression, somnolence, REM sleep behavior disorder (RBD), and autonomic nervous system symptoms were more frequently reported among high‐self‐reporters. (Further details are provided in Appendix S1.)

For scales with clinician report based on patient information, findings were mixed. In MDS‐UPDRS IA, the most complex behavior was observed in high‐self‐reporters, followed by intermediate‐ and low‐self‐reporters, as described earlier. On the Schwab and England Activities of Daily Living (ADL), intermediate‐self‐reporters showed higher functionality compared to both low‐ and high‐self‐reporters, whereas no difference between low‐ and high‐self‐reporters was found (92.8 ± 5.5 vs. 94.2 ± 5.6 vs. 91.4 ± 6.5, in low‐, intermediate‐, and high‐self‐reporters, respectively, *P* < 0.001).

By contrast, the three groups did not differ on most clinician‐examined and objective measures (other than MDS‐UPDRS III), except for the HY stage and University of Pennsylvania smell identification test (UPSIT) score. Low‐self‐reporters were in higher HY stages compared to intermediate‐ and high‐self‐reporters, whereas no difference between intermediate‐ and high‐self‐raters was found (1.8 ± 0.4 vs. 1.5 ± 0.5 vs. 1.5 ± 0.5, in low‐, intermediate‐, and high‐self‐reporters, respectively, *P* < 0.001). On UPSIT olfactory test, high‐self‐reporters had the best performance compared to low‐ and intermediate‐self‐reporters. Intermediate‐ and low‐self‐reporters did not differ from each other (21.8 ± 7.7 vs. 21.7 ± 7.9 vs. 24.1 ± 9.1, in low‐, intermediate‐, and high‐self‐reporters, respectively, *P* = 0.044). Despite higher patient‐reported orthostatic symptoms among high‐self‐reporters versus low‐ and intermediate‐self‐reporters (MDS‐UPDRS IB, light headedness on standing, *P* = 0.011), the objectively measured systolic blood pressure drop was not statistically different between the groups (*P* = 0.6).

There were no differences in self‐reported cognitive impairment according to MDS‐UPDRS IA between groups (*P* = 0.3). Similarly, there was no difference in performance on any cognitive test (all *P* > 0.05; Table 2).

Finally, neither DaT‐SPECT nor CSF biomarkers revealed any differences between low‐, intermediate‐, and high‐self‐reporters (all *P* > 0.05; Table 2).

## PASADENA

In the PASADENA cohort, mean baseline age was 59.9 ± 9.1 years, and 67.4% were men. Three hundred and sixteen participants were grouped in 80 low‐self‐reporters, 157 intermediate‐self‐reporters, and 79 high‐self‐reporters. There were no group differences in sociodemographic or disease‐related characteristics (all *P* > 0.05; Table 1).

Regarding patient‐reported measures, MDS‐UPDRS part I score was lowest in low‐self‐reporters, followed by intermediate‐self‐reporters and high‐self‐reporters (2.8 ± 2.2 vs. 4.3 ± 3.3 vs. 7.1 ± 4.8, in low‐, intermediate‐, and high‐self‐reporters, respectively, *P* < 0.001). The same pattern was observed when subdivided into parts IA and IB (part IA: 0.7 ± 1.0 vs. 1.0 ± 1.3 vs. 1.9 ± 2.3, in low‐, intermediate‐, and high‐self‐reporters, respectively, *P* < 0.001; part IB: 2.1 ± 1.8 vs. 3.3 ± 2.4 vs. 5.2 ± 3.4, in low‐, intermediate‐, and high‐self‐reporters, respectively, *P* < 0.001) (details of item level are provided in Appendix S1).

Patient‐reported scores of depression and anxiety, RBD, Parkinson's Disease Questionnaire (PDQ‐39) score, and autonomic symptoms were higher in high‐self‐reporters (details in Appendix S1).

For clinician‐report scales based on patient information, highest impairment of MDS‐UPDRS IA was noted in high‐self‐reporters, followed by intermediate‐ and low‐self‐reporters. In the Schwab and England ADL scale, only high‐self‐reporters differed from the other groups, whereas no differences were observed between low‐ and intermediate‐raters (92.8 ± 5.9 vs. 93.7 ± 6.2 vs. 90.0 ± 5.8, in low‐, intermediate‐, and high‐self‐reporters, respectively, *P* < 0.001).

The only clinician‐examined measure that was analyzed in the PASADENA cohort (other than MDS‐UPDRS III) was HY stage, on which high‐self‐reporters were in lower stages compared to intermediate‐ and low‐self‐reporters (2.0 ± 0.2 vs. 1.7 ± 0.5 vs. 1.6 ± 0.5, in low‐, intermediate‐, and high‐self‐reporters, respectively, *P* = 0.0005).

### Intercorrelation between Different Parts of MDS‐UPDRS

In PPMI, MDS‐UPDRS II scores correlated weakly positively with MDS‐UPDRS part IA (r_s_ = 0.24, *P* < 0.001), moderately with MDS‐UPDRS part III (r_s_ = 0.38, *P* < 0.001), and strongly with MDS‐UPDRS part IB (r_s_ = 0.54, *P* < 0.001). The MDS‐UPDRS III correlated weakly positively with MDS‐UPDRS parts IA (r_s_ = 0.11, *P* = 0.027) and IB (r_s=_0.27, *P* < 0.001) scores.

**FIG. 2:**
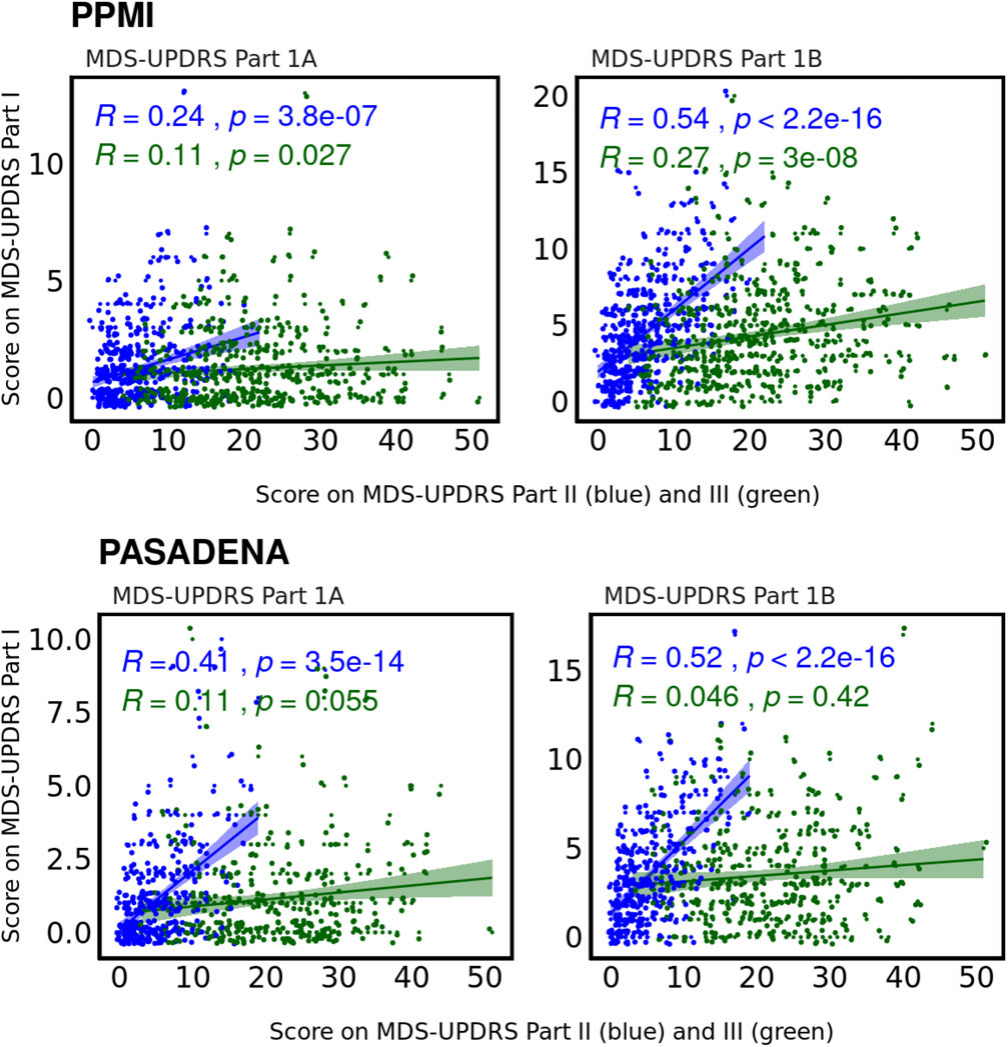
Spearman's correlations show positive associations of MDS‐UPDRS (Movement Disorder Society Unified Parkinson's Disease Rating Scale) parts IA (left panel) and IB (right panel) with MDS‐UPDRS part II and part III.

In PASADENA, MDS‐UPDRS II correlated weakly positively with MDS‐UPDRS part III (r_s_ = 0.28, *P* < 0.0001), moderately with MDS‐UPDRS part IA (r_s_ = 0.41, *P* < 0.0001), and strongly with MDS‐UPDRS part IB (r_s_ = 0.52, *P* < 0.0001). There were no significant associations between MDS‐UPDRS part III and either MDS‐UPDRS parts IA or IB scores (both r_s_ < 0.11, *P* > 0.05). Correlation plots are shown in Figure 2.

### Predictors of MDS‐UPDRS Part II and Part III Scores

In PPMI, a multivariate regression model revealed that MDS‐UPDRS part II scores were predicted by MDS‐UPDRS part IB (β = 0.45, 95% confidence interval [CI] = [0.37, 0.54]) and part III (β = 0.27, 95% CI = [0.19, 0.35]) scores. MDS‐UPDRS part IA, age, and sex were not predictors of part II.

Also, among the aforementioned variables, only MDS‐UPDRS part II (β = 0.36, 95% CI = [0.25, 0.46]) and age (β = 0.02, 95% CI = [0.01, 0.03]) predicted part III scores.

In PASADENA, among the predictor variables MDS‐UPDRS part III, part IA, part IB, age, and sex, MDS‐UPDRS part III (β = 0.22, *P* < 0.001), MDS‐UPDRS part IA (β = 0.26, *P* < 0.001), and MDS‐UPDRS part IB (β = 0.44, *P* < 0.001) significantly predicted MDS‐UPDRS part II scores. Based on the standardized beta coefficients, MDS‐UPDRS part IB was identified as the strongest predictor of MDS‐UPDRS part II, followed by part IA. Neither age nor sex significantly predicted MDS‐UPDRS part II scores. MDS‐UPDRS part III was predicted by part II (β = 0.36, *P* < 0.001) and sex, with lower scores for women compared to men (β = −0.13, *P* = 0.022). MDS‐UPDRS part IA, part IB, and age were not predictive of MDS‐UPDRS part III scores.

Summarized findings of the two cohorts are reported in Appendix S1.

## Discussion

This study examined the relationship between the degree of self‐reported motor‐related function impairment and clinician‐examined motor sign severity, as evaluated by discrepancies between scores on MDS‐UPDRS parts II and III. We found consistent response patterns across variables and two independent cohorts, such that individuals who endorsed a high number and/or severity of experienced motor symptoms (part II) also endorsed a greater number of nonmotor symptoms across the spectrum of patient‐reported questionnaires. However, when compared to clinician‐examined ratings, point estimates tended to go in the opposite direction, such that high‐self‐reporters tended to have less severe clinician‐examined objective signs than low‐self‐reporters and vice versa. Scores of intermediate self‐reporters were in the intermediate range on most measures. The results were consistent across variables and showed similar patterns in both PPMI and PASADENA populations.

In the PASADENA study, we found that MDS‐UPDRS II—which is intended to measure patients’ judgments of motor‐related impact in daily life—is predicted not only by clinician‐examined motor sign severity on part III but also numerically even more strongly by nonmotor symptoms reported in parts IA and IB. In the PPMI study, part IB was also the strongest predictor of part II, followed by part III scores (findings are reported in Appendix S1).

There are likely two primary (and not mutually exclusive) explanations for these findings.

The first explanation reflects the idea that “motor” activities do not exclusively measure motor behavior. For example, difficulties doing hobbies and with dressing or with hygiene activities may be caused directly by motor impairment but also could be caused by impaired cognition, fatigue, pain, and so on. This potential association is currently not considered how most of the items in MDS‐UPDRS part II are phrased; that is, for a participant responding to the questionnaire it is not always clear that the item should assess the contribution of motor problems only. Moreover, some individuals might find it difficult to distinguish between the sources of their problems. Similar issues were also observed in part II's predecessor scale, the UPDRS‐ADL.^18^ Therefore, the discriminant validity of MDS‐UPDRS part II as a scale to measure pure motor aspects of experiences of daily living is questionable and subject to jingle‐jangle fallacies (ie, the name of a scale drives how it is interpreted). If MDS‐UPDRS part II has stronger correlations with nonmotor symptoms than with motor symptoms, then it may not only be measuring what it was designed to measure.

The second explanation is the influence of response tendencies such that patients who self‐rate motor‐related symptoms higher than their motor examination findings also consistently self‐report a greater severity/impairment across a diverse array of PD symptoms, despite no worsening of examination findings or objective tests. Indeed, response style biases could exist in patient‐reported (or any self‐reported) questionnaires, meaning that individuals endorse symptoms in a certain way regardless of the objective severity or content of the question.^19^ This is a phenomenon that has been documented outside the field of PD. According to the Symptom Perception Hypothesis, patients with negative affection tend to report more physical health issues.^2^, ^3^ In particular, it has previously been shown that depression was associated with recalling more physical symptoms, whereas anxiety was associated with reporting more momentary physical symptoms.^4^

Both explanations are supported by findings in previous studies. Indeed, treatment of depression was associated with improved self‐reported quality of life and less self‐reported functional motor‐related impact on the UPDRS‐ADL score but no change in the UPDRS motor score.^20^ Weintraub and colleagues^8^ also reported that nonmotor symptoms (ie, signs of depression or cognitive impairment), but not physician‐assessed motor sign severity, were significant predictors of functional disability in PD as measured by the (self‐reported) UPDRS‐ADL subscale. Others have reported positive correlations between depression and higher self‐reported functional disability, motor impairment, and disease severity.^21^ These findings could support the first explanation; for example, someone who suffers from depression may perform daily (motor) activities slowly or with more difficulty, due to apathy, subjective fatigue, and so on In line with the second explanation, depression may also change to what degree patients recognize, perceive, or are impacted by any motor difficulties that make any activities more difficult.

In addition to psychiatric comorbidities, data collection method, the language of the questionnaire, and cognitive load, characteristics of respondents such as age, sex, education, income, race, culture, and personality are among the variables that can affect response style.^19^ However, in the present analyses we did not find any differences in the assessed demographic variables between groups, which may, in part, be due to the restricted range of demographic and cultural variables in these populations. It is important to note that most of the self‐reported scales used in the present studies are intercorrelated, driven by the partially overlapping (or even the same) constructs being assessed. The pattern that high‐self‐reporters reported more symptoms overall indicates that the patients were consistent with how they reported their symptoms.

The present results have implications for quantifying treatment response over time, both in the clinic and in clinical trials. If response tendencies, other nonmotor symptoms, and/or how a question is formulated drive higher values on a self‐reported “motor‐related” scale, this needs to be considered when interpreting findings. This consideration is instinctive for experienced clinicians; for example, if a major negative life event occurs in a patient who also reports more motor symptoms, clinicians consider the emotional state of the patient rather than automatically increasing levodopa doses. However, when individualized evaluation is impossible, as in clinical trials, important bias can occur. In particular, whereas a stable response pattern could potentially be adjusted for in trial design, any change in this effect during the trial can be problematic. One possible approach to potential bias may be to correct self‐reported measures like MDS‐UPDRS part II for potential effects of noteworthy nonmotor symptoms, for instance, by a regression‐based approach, similar to what is done in normative studies that correct cognitive scales for potential sociodemographic effects like level of education. Further research is required to better understand what drives the present results and the utility of such an approach.

### Study Limitations

There are some limitations to this study. First, we analyzed data from individuals with early PD, which were marked by low levels of impairment, particularly in MDS‐UPDRS parts I and II; therefore, there may be a limited dynamic range. The findings highlight the immediate need for patient‐reported outcome measures, which measure symptoms that meaningfully impact patients’ lives in the earliest stages of the disease. The importance of patient‐reported outcomes (ie, MDS‐UPDRS parts I and II) may be higher in a population with more advanced disease stages. Only 4% of the PASADENA population showed abnormal scores of anxiety and depression at baseline, whereas, in the PPMI, 14% of the whole sample showed signs of depression (Geriatric Depression Scale ≥5), and 23.8% showed signs of anxiety (State–Trait Anxiety Inventory >75th percentile). Nevertheless, the observed results were similar between PPMI and PASADENA. It is possible that individuals with depression/functional impairment might be less likely to participate in a research study or clinical trial, leading to an underrepresentation of the role of depression or anxiety in our study. Moreover, our approach of assigning individuals to groups of low‐, intermediate‐, and high‐self‐reporters has limitations. We based the classification on the difference between normalized MDS‐UPDRS part II and part III scores and labeled the lower and upper 25% of all individuals as low‐ and high‐self‐reporters, respectively, for each population separately. Thus, the classification is based on the distribution of the difference between part II and part III in the respective population and is therefore highly sample dependent. The results may differ if replicated in another study population. Nevertheless, we tested this approach in two independent samples (PPMI and PASADENA) and achieved overall consistent results, which corroborates the findings.

Future directions are explained in Appendix S1.

## Conclusions

Self‐reported motor‐related impact in daily life, as assessed by MDS‐UPDRS II, not only reflects examination‐based severity (ie, MDS‐UPDRS III) but is also strongly associated with nonmotor symptoms. This may reflect both a direct impact of nonmotor manifestations on motor function and differences in response tendencies to self‐reported questionnaires. These factors must be considered when using self‐reported rating scales such as MDS‐UPDRS II in clinical research and drug trials.

## Author Roles

(1) Research project: A. Conception, B. Organization, C. Execution; (2) Statistical analysis: A. Design, B. Execution, C. Review and critique; (3) Manuscript preparation: A. Writing of the first draft, B. Review and critique.

S.Z.: 1A, 1B, 1C, 2A, 2B, 3A

A.E.T.: 1A, 1B, 1C, 2A, 2B, 3A

N.L.: 1C, 2B, 3A

D.T.: 1B, 2C, 3B

F.L.: 1B, 2C, 3B

G.P.: 1B, 2C, 3B

K.I.T.: 1A, 1B, 2A, 2C, 3B

R.B.P.: 1A, 1B, 2A, 2C, 3B

## Supporting information


**APPENDIX S1.** Supporting information

## Data Availability

The PPMI data that support the findings of this study are openly available in www.ppmi-info.org. The PASADENA data are not publicly available due to privacy or ethical restrictions. Data can be available on request from authors affiliated with Roche.
