# Supplementary material for: Self‐Report versus Clinician Examination in Early Parkinson's Disease
Source: Mov Disord. 2021 Dec 13;37(3):585–97. doi: 10.1002/mds.28884 (PMC9299700; doi:10.1002/mds.28884)
Supplement: Supplementary file 1 — APPENDIX S1. Supporting information [file MDS-37-585-s001.pdf]

Self-report versus clinician-examination in early Parkinson's disease

SUPPLEMENTARY MATERIALS:

- ✓ **Details of the Measured Variables:**

  a) Sociodemographic characteristics
  b) Disease characteristics
  c) Patient-reported measures
  d) Clinician-reported measures based on patient-reported information
  e) Clinician-examined and objective measures
  f) Cognitive tests
  g) DaT-SPECT
  h) CSF biomarkers

- ✓ **Methods - Additional Details on Statistical Analysis**
- ✓ **Results - Additional Details**
- ✓ **Post-Hoc Analysis**
- ✓ **Summary of Findings**
- ✓ **Other indices to categorize patients**
- ✓ **Future Directions**
- ✓ **Supplementary Materials References**

Details of the Measured Variables:

The protocol, study design, sampling, and data collection of PPMI can be found on the PPMI website at https://www.ppmi-info.org/study-design/research-documents-and-sops (1). The protocol and inclusion criteria of PASADENA are also provided at https://clinicaltrials.gov/ct2/show/NCT03100149.

Sociodemographic, disease-characteristics, other patient-reported and clinician-examined measures, cognitive outcomes, and biomarkers:

We compared low- vs. intermediate- vs. high-self-reporters with respect to:

a) Sociodemographic characteristics:

We evaluated sex (in two categories: male/female), age (calculated based on the date of birth, by year), race (identify self as White/Black/Asian/other), years of education, dominant hand (right/left/ambidexterity). Height and weight were measured at the time of the baseline interview, and body mass index (BMI) was calculated using the formula: BMI=$\frac{Weight\ (kg)}{Height\ (m)^2}$.

b) Disease characteristics:

We measured age of onset (by year), duration of disease (by month), the side most affected at PD onset (right/left/symmetric), existence of first-degree family with PD (yes/no). Genetic variants were evaluated based on the number of cases who were carriers of β-glucocerebrosidase (GBA) or Leucine-rich repeat kinase 2 (LRRK2) gene mutation markers and prior MAO-B inhibitor therapy (yes/no).

c) Patient-reported measures:

MDS-UPDRS part IB and part II

Part IB consists of 7 questions about non-motor aspects of experiences of daily living; see Table 2 in main manuscript. Together with part IA (see clinician-reported measures), part IB is summed up to part I. Part II has 13 questions about the motor experiences of PD patients in their daily lives. All of the questions are ranked on a 5-Likert scale from 0 to 4. So, the range of total score for either of parts I or II varies between 0 and 52.

Anxiety in the PPMI was evaluated using the State-Trait Anxiety Inventory (STAI). It has 40 questions, including 20 questions on current condition (state) and 20 questions on long-term condition (trait). Each question is measured in 4-Likert from 1 to 4 (range of score: 40-160). The validity and reliability of the questionnaire are reported elsewhere (2).

Depression in PPMI was assessed through the short form of the Geriatric Depression Scale (GDS-15). It consists of 15 yes/no questions (range of score: 0-15). The cut-off ≥5 has a sensitivity and specificity of 71.8% and 78.2%, respectively. It is considered a useful screening tool for depression among the elderly (3).

Anxiety and depression in PASADENA were calculated using the Hospital Anxiety and Depression Scale (HADS). It is a 14-item questionnaire, 7 questions are related to anxiety (HADS-A), and 7 questions measure depression (HADS-D). Each question was measured on a 4-Likert scale from 0 to 3, where 0 = no symptom of anxiety/depression, and 3 = highest level of anxiety/depression (range of score for each section: 0-21). Score≥11 for each section was considered abnormal.

Epworth sleepiness scale (ESS) was used to evaluate the risk of daytime sleepiness. It evaluates the chance of dozing in 8 different daily activities/situations. Each question is

ranked on a 4-Likert scale from 0 to 3, where 0 = would never doze and 3 = high chance of dozing (range of score: 0-24). The validity and reliability of this scale were previously reported (4, 5).

Impulse control in PPMI was evaluated using the questionnaire for impulsive-compulsive disorders in Parkinson's disease (QUIP). QUIP score can be effectively used to screen for impulsive-compulsive disorders in PD patients (6). It has three sections: impulsive control (gambling, sex, buying, eating), compulsive behaviors (punding, hobbies, walking), and medication use. Being positive for any of the sub-domains of impulsive or compulsive behaviors result in a score of 1, such that the total score ranges from 0 to 7.

REM sleep behavior disorder screening questionnaire (RBDSQ) was used to screen for RBD. It is an approved screening tool with 10 items with a score range from 0 (no symptoms) to 13 (all symptoms endorsed) (7).

Autonomic dysfunction during the previous month was measured with scales for outcomes in Parkinson's disease-autonomic questionnaire (SCOPA-AUT). It includes 6 sub-domains: 7 questions on the gastrointestinal system, 6 questions related to the urinary system, 3 questions for cardiovascular, 4 questions for the thermoregulatory system, 1 question on pupillomotor, and 2 questions on sexual dysfunction. Each question can be scored on a 4-point Likert scale where 0 = never having the problem and 3 = often having the problem, such that the total SCOPA-AUT score ranges from 0 to 69. This questionnaire was validated in PD patients (8).

Parkinson's Disease Questionnaire 39 (PDQ-39) is a self-reported questionnaire to assess the impact of PD on patients' wellbeing and functioning during the last month. It has 39 questions in 8 domains: activities of daily living (6 questions), bodily discomfort (3

questions), cognition (4 questions), communication (3 questions), emotional wellbeing (6 questions), mobility (10 questions), social support (3 questions), and stigma (4 questions). Lower scores indicate better perceived health status. Each scale is transformed to have a range from 0 (no problem at all) through to 100 (highest level of problem) with each scale being calculated as follows: scale score = the total of the raw scores of each item in the scale divided by the maximum possible raw score of all the items in the scale multiplied by 100 (9). Overall score is summarized as PDQ-39 Summary Index and is calculated as the sum of dimension total score divided by 8.

d) Clinician-reported measures based on patient-reported information:

MDS-UPDRS part IA has 6 questions related to non-motor experiences, specifically complex behaviors. All of the questions are ranked on a 5-Likert scale from 0 to 4.

The Modified Schwab and England Activities of Daily Living scale evaluates the functionality of PD patients and measures their ability to do daily activities independently (10).

e) Clinician-examined and objective measures:

Hoehn and Yahr scale, which was included in the MDS-UPDRS questionnaire, is a staging scale of PD. It is a reliable and valid tool that provides a global assessment of PD based on the pattern of involvement and disabilities (11).

MDS-UPDRS part III has 33 questions related to motor examinations. Each question is ranked on a 5-Likert scale from 0 (normal) to 4 (severe), with the total score range from 0 to 132. Among these questions, 10 items are related to postural, kinetic, and rest tremor

in different extremities and constancy of rest tremor. Furthermore, 5 items are related to rigidity in the neck or extremities. The summation of these respective scores results in the tremor score and rigidity subscores. Also, 11 items ask about the pace of movements, including finger tapping, hand movements, pronation-supination, toe-tapping, and leg agility for each side, as well as body bradykinesia. The summation of these scores resulted in the bradykinesia subscore.

Objective measures:

University of Pennsylvania smell identification test (UPSIT) is an accurate and reliable olfaction test consisting of 40 questions (12). Based on the score, participants can be categorized as anosmic (UPSIT≤18), hyposmic (18<UPSIT≤34 in men or 35 in women), and normosmic (UPSIT>34 in men or 35 in women) [PPMI only].

Drop in systolic blood pressure was calculated based on the difference between supine and standing systolic blood pressure, which was measured after 1-3 minutes of quiet rest or standing position, respectively.

f) Cognitive tests [PPMI only]

Montreal cognitive assessment (MoCA) is a validated test, highly sensitive for mild cognitive impairment in PD patients (13). MoCA score ranges from 0 to 30 (best performance), and it can screen different cognitive aspects such as visuospatial/executive, naming, memory, attention, language, abstraction, and time and place orientation (14). To adjust for the education, one point is awarded to patients with ≤12 years of education.

Benton judgment of line orientation test (BJLOT) is a valid and reliable test of visuospatial perception (15). The PPMI used the short form with 15 items and multiplied the scores by 2, so the range of scores could vary from 0 to 30. We used the adjusted score for age and education.

Hopkins verbal learning test-revised (HVLT-R) was used to assess different aspects of memory. Patients were given a list of words and were instructed to remember and recall as many as they can immediately (immediate recall) and after 20-25 minutes (delayed recall). Immediate recall test was performed in 3 trials, and the total number of recalled words over all three trials was defined as 'total recall'. The 'retention score' was defined as the number of items recalled at delayed recall divided by the maximum number of items recalled from the 2nd and 3rd immediate recall trials. 'Delayed recognition test' was performed immediately after the delayed recall test, whereby patients were given another list of words containing the words from the learning trial mixed with novel words and instructed to identify the words that had been previously presented. The number of words identified correctly, subtracted by the number of false positive, was used to define a 'recognition discrimination index'. For all of the mentioned scores, we analyzed the standardized T-score.

Letter number sequencing (LNS) test is a part of the Wechsler Adult Intelligence Scale (WAIS) intelligence test, which measures working memory capacity (16). Participants are given a group of numbers and letters, and they are asked to repeat the numbers first, in order, starting with the lowest number and then say the letters in alphabetical order. This test has 21 trials with a range of scores from 0 to 21. It starts with a combination of one number and one letter, and gradually becomes more difficult.

Semantic fluency test (SFT) screens for executive function and semantic memory. Patients are instructed to produce as many names of vegetables, fruits, and animals in three separate 60-second trials. They were awarded 1 point per each unique word produced. Thus, there is no pre-defined range of scores.

Symbol digit modality test (SDMT) measures the speed of processing and attention (17). Participants are given a list of 9 symbols with a number assigned to each symbol. Then they are given a list of symbols and they have to fill it with the paired number, as fast as possible, within 90 seconds and without skipping any symbol. The score ranges from 0 to 110. Here we used the standardized T-score.

g) DaT-SPECT [PPMI only]

Dopamine transporter single photon emission computed tomography (DaT-SPECT) with ^{123}I-ioflupane was performed in PPMI, and the striatal binding ratios (SBR) were calculated using the occipital lobe as the reference region. In this study, we compared the SBR of the lowest measured caudate, putamen, and entire striatum among the reported groups. The striatum was calculated as the mean of right and left caudate and putamen.

h) Cerebrospinal fluid biomarkers [PPMI only]

Lumbar puncture was performed for patients, and the levels of amyloid β 1-42, α-Synuclein protein, total and phosphorylated tau were measured in the cerebrospinal fluid.

Methods - Additional Details:

Statistical Analysis

Kruskal-Wallis non-parametric tests compared low-, intermediate-, and high-self-reporter scores on continuous variables. For variables that showed overall statistical significant group differences, post-hoc Dunn test was used to determine which pairs of groups differed from each other. Categorical variables were analyzed using Pearson's Chi-square test. No post-hoc tests were conducted for categorical variables.

Statistical analyses were performed by using SPSS v24 for the PPMI dataset and R version 3.5.3 (2019-03-11; R Foundation, Vienna, Austria) with Jupyter Notebook server 5.2.1 (running on Python 3.6.8, Anaconda custom [64-bit] 2018-12-30) for the PASADENA dataset.

Results - Additional Details:

PPMI and PASADENA: MDS-UPDRS parts II and III

We confirmed the categorization into relative low-, intermediate- and high-self-reporters based on the difference in average severity rating for MDS-UPDRS part II vs. III: In both cohorts, MDS-UPDRS II scores were indeed highest among high-self-reporters, followed by intermediate- and low-self-reporters (PPMI: 3.5±2.5 vs. 4.9±3.1 vs. 10.4±4.1 in low-, intermediate-, and high-self-reporters, respectively, $p<0.001$; PASADENA: 2.9±2.3 vs. 4.4±2.9 vs. 9.7±4.1 in low-, intermediate-, and high-self-reporters, respectively, $p<0.001$). Groups also differed with respect to clinician-examined MDS-UPDRS III scores, with low-self-reporters receiving the highest scores, followed by intermediate- and high-self-raters (PPMI: 28.1±8.0 vs. 18.6±8.0 vs. 18.4±7.4 in low-, intermediate-, and high-self-reporters, respectively, $p<0.001$; PASADENA: 29.1±6.1 vs. 19.4±8.0 vs. 17.8±8.9 in low-, intermediate-, and high-self-reporters, respectively, $p<0.001$). Note

that because self-reporter groups were categorized based on the difference between MDS-UPDRS parts II and III scores, the observed differences in raw parts II and III scores over groups are confounded: We instead concentrated on measures independent of MDS-UPDRS parts II and III.

PPMI:

Symptoms of anxiety and depression were more frequently reported among high-self-reporters (State-Trait Anxiety Inventory (STAI) score of 64.4±18.8 vs. 63.9±17.4 vs. 69.6±19.3 in low-, intermediate-, and high-self-reporters, respectively, p=0.022; Geriatric Depression Scale (GDS) score of 2.1±2.4 vs. 2.2±2.3 vs. 3.0±2.7 in low-, intermediate-, and high-self-reporters, respectively, p=0.005).

High-self-reporters reported greater somnolence (Epworth Sleepiness Scale: 5.2±3.0 vs. 5.6±3.5 vs. 6.9±3.7, in low-, intermediate-, and high-self-reporters, respectively, p=0.001), more REM sleep behavior disorder (RBDSQ: 3.2±2.2 vs. 4.1±2.7 vs. 5.1±2.9, in low-, intermediate-, and high-self-reporters, respectively, p<0.001), and more autonomic nervous system symptoms (scales for outcomes in Parkinson's disease-autonomic questionnaire (SCOPA-AUT): 7.2±4.3 vs. 9.3±6.3 vs. 12.3±6.6, in low-, intermediate-, and high-self-reporters, respectively, p<0.001).

PASADENA

All groups differed from each other on item level, with groups differences in cognitive impairment (p=0.0005), sleep problems (p<0.0001), daytime sleepiness (p=0.0004), and pain and other sensations (p=0.0001). High-self-reporters additionally reported more impairment compared to both low- and intermediate-self-reporters in depressed mood (p<0.001), apathy (p=0.0005), features of dopamine dysregulation syndrome (p=0.01), urinary problems (p=0.001),

lightheadedness on standing (p=0.01), and fatigue (p=0.0007). Low-self-reporters differed from both intermediate- and high-self-reporters in the item related to constipation problems (p=0.001).

Depression scores, assessed with HADS-D, differed across all groups (3.3±3.1 vs. 3.7±2.9 vs. 5.7±3.7 in low-, intermediate-, and high-self-reporters, respectively, p<0.0001). The remaining patient-reported scales only revealed differences for high-self-reporters compared to the other two groups, with highest scores in anxiety (HADS-A) (4.2±2.7 vs. 4.7±3.0 vs. 5.5±3.6 in low-, intermediate-, and high-self-reporters, respectively, p<0.001), RBDSQ (3.1±2.7 vs. 3.3±2.5 vs. 4.2±2.9 in low-, intermediate-, and high-self-reporters, respectively, p=0.005), and SCOPA-AUT (6.4±4.3 vs. 7.6±5.1 vs. 10.5±7.2, in low-, intermediate-, and high-self-reporters, respectively, p=0.0005).

Post-Hoc Analysis:

Post-hoc analyses on PPMI revealed that for almost all patient-reported measures, including those not directly related to the motor state, high-self-reporters endorsed more symptoms/impairment compared to low- and intermediate-self-reporters (Table 2).

The post-hoc analyses revealed fewer differences between individuals in the low- vs. intermediate-self-reporter group. Low-self-reporter differed from intermediate-self-reporter only in the modified Schwab and England Activities of Daily Living (ADL) scale (92.8±5.5 vs. 94.2±5.6 in low- and intermediate-self-reporters, respectively), the RBDSQ (3.2±2.2 vs. 4.1±2.7 in low- and intermediate-self-reporter, respectively), and the SCOPA-AUT (7.2±4.3 vs. 9.3±6.3 in low- and intermediate-self-reporter, respectively). No differences between those groups were found on other patient-reported scales.

Post-hoc analyses on PASADENA revealed a pattern of response, as such that high-self-reporters endorsed the most and low-self-reporters the least symptoms/impairment on almost all measures (Table 2).

Summary of Findings:

To summarize, in both cohorts, high-self-reporters, i.e., individuals who reported relatively more motor function impacts compared to clinical motor sign examination (part II>part III), reported worse conditions in most patient-reported measures across multiple non-motor domains compared to low-and intermediate-self reporters. In contrast, their scores on all motor or non-motor examination-based/objective measures were comparable to those in the low- and intermediate-self-reporter groups.

Contrary to this, in the group of low-self-reporters, individuals not only reported fewer and less severe motor symptoms relative to the clinical motor sign examination, but also reported fewer symptoms on other non-motor patient-reported scales compared to individuals in the intermediate- and especially the high-self-reporter group.

In general, post-hoc tests revealed differences between low- and high-self-reporters on almost all patient-reported measures tests. However, in the post-hoc comparisons, high-self-reporters showed more notable differences compared to intermediate-self-reporters than low-self-reporters compared to intermediate-self-reporters.

Other indices to categorize patients:

We chose one index (motor impact and impairment) to examine response tendencies, mainly because they contain both patient- and clinician-reported measures. This is not the only possible

direct patient/clinician pairing; we did not have available patient-reported measures of olfaction, objective measures of sleep, etc. However, in the PPMI dataset, we also explored direct correlations between individual motor-related measurements (e.g., tremor), as well as patient-reported cognition vs. neuropsychological test results (data not shown). Although analysis was limited by more variability/noise, we observed overall patterns similar to our motor analysis.

Stability over time:

To assess to what degree response patterns remain stable over time, we performed follow-up analysis on the PPMI database. Among high-self-reporters at baseline, 9.9% (8 individuals) became low-reporters at follow-up compared to 54.3% who remained high-reporters. Of low-self-reporters at baseline, 11.8% became high-self-reporters at follow-up (10 patients), vs. 42.4% who stayed in the same category. When stratifying follow-up results into top vs. bottom half of response categories, 77.8% of baseline 'high-self-reporters' remained in the top half of 'high-reporting' (chance=50%), and 70.8% of baseline 'low-self-reporters' remained in the bottom 'low-reporting' half. Therefore, whereas patients showed a consistent pattern of response tendencies, a substantial minority shifted response patterns.

Future Directions:

Whereas this study found that different response patterns exist in self-reported measures, we are not able to answer the underlying reason for these observations or deduct any causal direction of the observed relationships between different measures. The PPMI and PASADENA datasets were not collected with the aim to conduct the present analysis, and the results are therefore exploratory in nature. In future studies, one might consider to first collect parts II and III, and then

interview patients to better understand reasons for patient’s ratings or additionally administer scales measuring concepts orthogonal to symptoms under investigation to generate an independent measure of response tendencies. Follow-up analyses on longitudinal data may shed light on this and provide more insights on these response patterns and the relationship between MDS-UPDRS part II, part III, and non-motor symptoms, also in more advanced disease stages. Moreover, longitudinal analyses are required to understand whether response tendencies are associated with different disease trajectories, and whether they change with time or symptomatic treatment.

Supplementary Materials References

1. The Parkinson Progression Marker Initiative (PPMI). Prog Neurobiol. 2011;95(4):629-35.
2. Julian LJ**.** Measures of anxiety: State-Trait Anxiety Inventory (STAI), Beck Anxiety Inventory (BAI), and Hospital Anxiety and Depression Scale-Anxiety (HADS-A). Arthritis Care Res (Hoboken). 2011;63 Suppl 11:S467-72.
3. Marc LG, Raue PJ, Bruce ML**.** Screening Performance of the 15-Item Geriatric Depression Scale in a Diverse Elderly Home Care Population. The American Journal of Geriatric Psychiatry. 2008;16(11):914-21.
4. Johns MW**.** Reliability and Factor Analysis of the Epworth Sleepiness Scale. Sleep. 1992;15(4):376-81.
5. Hobson DE, Lang AE, Martin WRW, Razmy A, Rivest J, Fleming J**.** Excessive Daytime Sleepiness and Sudden-Onset Sleep in Parkinson DiseaseA Survey by the Canadian Movement Disorders Group. JAMA. 2002;287(4):455-63.
6. Weintraub D, Hoops S, Shea JA, Lyons KE, Pahwa R, Driver-Dunckley ED, et al. Validation of the questionnaire for impulsive-compulsive disorders in Parkinson's disease. Movement disorders : official journal of the Movement Disorder Society. 2009;24(10):1461-7.
7. Stiasny-Kolster K, Mayer G, Schäfer S, Möller JC, Heinzel-Gutenbrunner M, Oertel WH**.** The REM sleep behavior disorder screening questionnaire—A new diagnostic instrument. 2007;22(16):2386-93.
8. Visser M, Marinus J, Stiggelbout AM, Van Hilten JJ**.** Assessment of autonomic dysfunction in Parkinson's disease: the SCOPA-AUT. Mov Disord. 2004;19(11):1306-12.
9. Peto V, Jenkinson C, Fitzpatrick R, Greenhall R**.** The development and validation of a short measure of functioning and well being for individuals with Parkinson's disease. Quality of Life Research. 1995;4(3):241-8.

10. Bjornestad A, Tysnes O-B, Larsen JP, Alves G. Reliability of Three Disability Scales for Detection of Independence Loss in Parkinson's Disease. Parkinson's disease. 2016;2016:1941034-.
11. Martinez-Martin P, Skorvanek M, Rojo-Abuin JM, Gregova Z, Stebbins GT, Goetz CG, et al. Validation study of the hoehn and yahr scale included in the MDS-UPDRS. 2018;33(4):651-2.
12. Doty RL, Frye RE, Agrawal U. Internal consistency reliability of the fractionated and whole University of Pennsylvania Smell Identification Test. Perception & Psychophysics. 1989;45(5):381-4.
13. Hoops S, Nazem S, Siderowf AD, Duda JE, Xie SX, Stern MB, et al. Validity of the MoCA and MMSE in the detection of MCI and dementia in Parkinson disease. Neurology. 2009;73(21):1738-45.
14. Nasreddine ZS, Phillips NA, Bedirian V, Charbonneau S, Whitehead V, Collin I, et al. The Montreal Cognitive Assessment, MoCA: a brief screening tool for mild cognitive impairment. J Am Geriatr Soc. 2005;53(4):695-9.
15. Spencer RJ, Wendell CR, Giggey PP, Seliger SL, Katzel LI, Waldstein SR. Judgment of Line Orientation: an examination of eight short forms. Journal of clinical and experimental neuropsychology. 2013;35(2):160-6.
16. Mielicki MK, Koppel RH, Valencia G, Wiley J. Measuring working memory capacity with the letter–number sequencing task: Advantages of visual administration. 2018;32(6):805-14.
17. Weintraub D, Simuni T, Caspell-Garcia C, Coffey C, Lasch S, Siderowf A, et al. Cognitive performance and neuropsychiatric symptoms in early, untreated Parkinson's disease. 2015;30(7):919-27.
